# *Mycobacterium tuberculosis* H37Rv: *In Silico* Drug Targets Identification by Metabolic Pathways Analysis

**DOI:** 10.1155/2014/284170

**Published:** 2014-02-25

**Authors:** Asad Amir, Khyati Rana, Arvind Arya, Neelesh Kapoor, Hirdesh Kumar, Mohd Asif Siddiqui

**Affiliations:** Department of Biotechnology, Meerut Institute of Engineering and Technology, N.H. 58, Delhi-Roorkee Highway, Baghpat Road Bypass Crossing, Meerut 250005, India

## Abstract

*Mycobacterium tuberculosis* (Mtb) is a pathogenic bacteria species in the genus *Mycobacterium* and the causative agent of most cases of tuberculosis. Tuberculosis (TB) is the leading cause of death in the world from a bacterial infectious disease. This antibiotic resistance strain lead to development of the new antibiotics or drug molecules which can kill or suppress the growth of *Mycobacterium tuberculosis*. We have performed an *in silico* comparative analysis of metabolic pathways of the host *Homo sapiens* and the pathogen *Mycobacterium tuberculosis* (H37Rv). Novel efforts in developing drugs that target the intracellular metabolism of *M. tuberculosis* often focus on metabolic pathways that are specific to *M. tuberculosis*. We have identified five unique pathways for *Mycobacterium tuberculosis* having a number of 60 enzymes, which are nonhomologous to *Homo sapiens* protein sequences, and among them there were 55 enzymes, which are nonhomologous to *Homo sapiens* protein sequences. These enzymes were also found to be essential for survival of the *Mycobacterium tuberculosis* according to the DEG database. Further, the functional analysis using Uniprot showed involvement of all the unique enzymes in the different cellular components.

## 1. Introduction

*Mycobacterium tuberculosis* (Mtb), the causative agent of tuberculosis (TB), remains a major health threat. Each year, 8 million new TB cases appear and 2 million individuals die of TB [1]. Further, about half a million new multidrug resistant TB cases are estimated to occur every year [2]. The existing drugs, although of immense value in controlling the disease to the extent that is being done today, have several shortcomings, the most important of them being the emergence of drug resistance rendering even the front-line drugs inactive. In addition, drugs such as rifampicin have high levels of adverse effects making them prone to patient incompliance. Another important problem with most of the existing antimycobacterials is their inability to act upon latent forms of the bacillus. In addition to these problems, the vicious interactions between the HIV (human immunodeficiency virus) and TB have led to further challenges for antitubercular drug discovery [3].

Recently, genome-scale metabolic network reconstructions for different organisms have enabled systematic analyses of metabolic functions and predictions of metabolism-related phenotypes. By collecting all possible biochemical reactions for specific organisms, different groups have reconstructed metabolic networks for bacteria, for example, *Escherichia coli, Helicobacter pylori,* and *Chromohalobacter salexigens*, eukaryotic microorganisms, mice, and even humans [4–6]. The website of the Systems Biology Research Group at the University of California, San Diego (http://gcrg.ucsd.edu/), provides a continuously updated list of genome-scale metabolic network reconstructions. Analysis of metabolic networks can provide insights into an organism's ability to grow under specific conditions. For example, given a specific set of nutrient conditions, flux balance analysis (FBA) of metabolic networks can accurately predict microbial cellular growth rates. In a recent work, a group of researchers used an approximate representation of in-host nutrient availability inferred from the literature to simulate the in-host metabolism of *Salmonella typhimurium* [7]. Moreover, metabolic network analyses can then be used to identify organism-specific essential genes by predicting the attenuation of microbial growth of specific deletion mutants [8–10].

The computational approach has been used to investigate novel drug targets in other pathogenic organisms such as *Pseudomonas aeruginosa* and in *Helicobacter pylori* [5, 11].

As most currently known, antibacterials are essentially inhibitors of certain bacterial enzymes; all enzymes specific to bacteria can be considered as potential drug targets [12]. In this study, we have adopted a strategy for comparative metabolic pathway analysis to find out some potential targets against *M. tuberculosis* (H37Rv). Only those enzymes which show unique properties than the host were selected as the target. Metabolic genes that are essential for pathogen growth but are not present in humans constitute actual and potential drug targets.

## 2. Materials and Methods

KEGG (Kyoto Encyclopedia of Gene and Genome) (http://www.genome.jp/pathways.html) [13] pathway database was used as a source of metabolic pathway information. Metabolic pathway identification numbers of the host *H. sapiens* and the pathogen *M. tuberculosis *(*H37Rv*) were extracted from the KEGG database. Pathways which do not appear in the host but are present in the pathogen according to KEGG database have been identified as pathways unique to *M. tuberculosis* as in comparison to the host *H. sapiens*. Enzymes in these unique pathways as well as enzymes involved in other metabolic pathways under carbohydrate metabolism, energy metabolism, lipid metabolism, nucleotide metabolism, amino acid metabolism, metabolism of other amino acids, and glycan biosynthesis were identified from the KEGG database. The corresponding protein sequences of enzymes involved in unique pathways were identified and their protein sequences were retrieved in FASTA format from KEGG database.

The unique enzymes were further analyzed for essentiality to pathogen by DEG (Database of Essential Genes) database (http://tubic.tju.edu.cn/deg/) [14], and considered cutoff score was >100 to enhance the specificity of enzyme in *M. tuberculosis.*

The obtained targets genes were further analyzed by UniProt (Universal Protein Resource) (http://www.uniprot.org/) database to find out their functions. This is required to find out the surface membrane proteins which could be probable vaccine targets.

## 3. Results and Discussion

### 3.1. Identification of Unique Pathways and Potential Drug Targets

Tuberculosis (TB) is a major cause of illness and death worldwide, especially in Asia and Africa. Globally, 9.2 million new cases and 1.7 million deaths from TB occurred in 2006, of which 0.7 million cases and 0.2 million deaths were in HIV-positive people [2]. The existing drugs have several shortcomings, the most important of them being the emergence of drug resistance.

No new anti-Mtb drugs have been developed for well over 20 years. In view of the increasing development of resistance to the current leading anti-Mtb drugs, novel strategies are desperately needed to avert the “global catastrophe” forecast by the WHO (World Health Organization). Therefore, computational approach for drug targets identification, specifically for *Mtb*, can produce a list of reliable targets very rapidly. These methods have the advantage of speed and low cost and, even more importantly, provide a systems view of the whole microbe at a time. Since it is generally believed that the genomes of bacteria contain genes both with and without homologues to the human host. Using computational approach for target identification it is very quick to produce a desirable list.

In the present study, 5 unique pathways, C5-branched dibasic acid metabolism, carbon fixation pathways in prokaryotes, methane metabolism, lipopolysaccharide biosynthesis, and peptidoglycan biosynthesis with 60 new nonhomologous targets were identified through *in silico* comparative metabolic pathway analysis of *Homo sapiens* and *M. tuberculosis H37Rv *using KEGG database. Pathways which are not present in the *Homo sapiens* but present in the *Mycobacterium* are designated as unique pathways. Design and targeting inhibitors against these nonhomologous sequences could be the better approach for generation of new drugs. Thus total 5 unique metabolic pathways have been taken in *M. tuberculosis* (Table 1).

### 3.2. Identification of Essential Genes

Essential genes are those indispensable for the survival of an organism, and their functions are, therefore, considered a foundation of life. Total 55 enzymes out of all were found to be essential for *M. tuberculosis* life cycle (Table 2). These targets were found to be potential targets and could be considered for rational drug design. Using metabolic pathway information as the starting point for the identification of potential targets has its advantages as each step in the pathway is validated as the essential function for the survival of the bacterium.

### 3.3. Identification of Drug Target's Functions Using UniProt

The subcellular localization analysis of all supposed essential and unique enzymes of *M. tuberculosis* were evaluated by UniProt server. As it was suggested that, membrane associated protein could be the better target for developing vaccines. After functional analysis unique enzymes involved in cellular components like cell wall, cytoplasm, extracellular region, plasma membrane, and so forth, their biological processes and their functions have been retrieved (Table 3).

In conclusion, the computational genomic approach has facilitated the search for potential drug targets against *M. tuberculosis.* Use of the DEG database is more efficient than conventional methods for identification of essential genes and it facilitates the exploratory identification of the most relevant drug targets in the pathogen. The current study can be carried forward to design a drug that can block these drug targets. The microorganisms are fast in gaining resistance to the existing drugs, so designing better and effective drugs needs a faster method.

## Figures and Tables

**Table 1:** Unique pathways of *M. tuberculosis * when compared to *H. sapiens*.

S. no.	Pathway name	Human	*Mycobacterium tuberculosis* H37Rv
1	Carbohydrate Metabolism		
1.1	C5-Branched dibasic acid metabolism	Absent	**Present**
2	Energy Metabolism		
2.1	Photosynthesis	Absent	Absent
2.2	Carbon fixation pathways in prokaryotes	Absent	**Present**
2.3	Methane metabolism	Absent	**Present**
3	Lipid Metabolism		
3.1	Fatty acid elongation in mitochondria	Present	Absent
3.2	Sphingolipid metabolism	Present	Absent
3.3	Arachidonic acid metabolism	Present	Absent
4	Nucleotide Metabolism	All Present	All Present
5	Amino Acid Metabolism	All Present	All Present
6	Metabolism of Other Amino Acids	All Present	All Present
6.1	Phosphonate and phosphinate metabolism	Absent	Absent
7	Glycan Biosynthesis and Metabolism		
7.1	N-Glycan biosynthesis	Present	Absent
7.2	Various types of N-glycan biosynthesis		Absent
7.3	Mucin type O-Glycan biosynthesis	Present	Absent
7.4	Other types of O-glycan biosynthesis	Present	Absent
7.5	Glycosaminoglycan biosynthesis—chondroitin sulfate	Present	Absent
7.6	Glycosaminoglycan biosynthesis—heparan sulfate	Present	Absent
7.7	Glycosaminoglycan biosynthesis—keratan sulfate	Present	Absent
7.8	Glycosaminoglycan degradation	Present	Absent
7.9	Glycosylphosphatidylinositol (GPI)-anchor biosynthesis	Present	Absent
7.10	Glycosphingolipid biosynthesis—lacto and neolacto series	Present	Absent
7.11	Glycosphingolipid biosynthesis—globo series	Present	Absent
7.12	Glycosphingolipid biosynthesis—ganglio series	Present	Absent
7.13	Lipopolysaccharide biosynthesis	Absent	**Present**
7.14	Peptidoglycan biosynthesis	Absent	**Present**
7.15	Other Glycan degradation	Present	Absent

**Table 2:** Essential enzymes using DEG.

S. no.	Entry no.	Protein name	Essential enzyme
1.	Rv1820	Acetolactate synthase	Yes
2.	Rv0951	Succinyl-CoA synthetase subunit beta	Yes
3.	Rv2987c	Isopropylmalate isomerase small subunit	Yes
4.	Rv1475c	*Aconitate hydratase *(EC: 4.2.1.3)	Yes
5.	Rv0066c	Isocitrate dehydrogenase (EC: 1.1.1.42)	Yes
6.	Rv2454c	2-Oxoglutarate ferredoxin oxidoreductase subunit beta (EC: 1.2.7.3)	Yes
7.	Rv1240	Malate dehydrogenase (EC: 1.1.1.37)	Yes
8.	Rv1098c	Fumarate hydratase (EC: 4.2.1.2)	Yes
9.	Rv0247c	Fumarate reductase iron-sulfur subunit (EC: 1.3.99.1)	Yes
10.	Rv3356c	Bifunctional 5,10-methylene-tetrahydrofolate dehydrogenase/5,10-methylene-tetrahydrofolate Cyclohydrolase (EC: 1.5.1.5 3.5.4.9)	Yes
11.	Rv0951	Succinyl-CoA synthetase subunit beta (EC: 6.2.1.5)	Yes
12.	Rv0904c	Putative acetyl-coenzyme A carboxylase carboxyl transferase subunit beta (EC: 6.4.1.2)	Yes
13.	Rv0973c	Acetyl-/propionyl-coenzyme A carboxylase subunit alpha (EC: 6.3.4.14)	Yes
14.	Rv1492	Methylmalonyl-CoA mutase small subunit (EC: 5.4.99.2)	Yes
15.	Rv3667	Acetyl-CoA synthetase (EC: 6.2.1.1)	Yes
16.	Rv0409	Acetate kinase (EC: 2.7.2.1)	Yes
17.	Rv0408	Phosphate acetyltransferase (EC: 2.3.1.8)	Yes
18.	Rv0243	Acetyl-CoA acetyltransferase (EC: 2.3.1.9)	Yes
19.	Rv0860	Fatty oxidation protein FadB	Yes
20.	Rv3667	Acetyl-CoA synthetase (EC: 6.2.1.1)	Yes
21.	Rv0373c	Carbon monoxyde dehydrogenase large subunit (EC: 1.2.99.2)	No
22.	Rv2900c	Formate dehydrogenase H (EC: 1.2.1.2)	No
23.	Rv1023	Phosphopyruvate hydratase (EC: 4.2.1.11)	Yes
24.	Rv1240	Malate dehydrogenase (EC: 1.1.1.37)	Yes
25.	Rv0070c	Serine hydroxymethyltransferase (EC: 2.1.2.1)	Yes
26.	Rv2205c	Hypothetical protein	Yes
27.	Rv0761c	Zinc-containing alcohol dehydrogenase NAD dependent AdhB (EC: 1.1.1.1)	Yes
28.	Rv0489	Phosphoglyceromutase (EC: 5.4.2.1)	Yes
29.	Rv0363c	Fructose-bisphosphate aldolase (EC: 4.1.2.13)	Yes
30.	Rv2029c	Phosphofructokinase PfkB (phosphohexokinase) (EC: 2.7.1.—)	Yes
31.	Rv1908c	Catalase-peroxidase-peroxynitritase T KatG (EC: 1.11.1.6)	Yes
32.	Rv0070c	Serine hydroxymethyltransferase (EC: 2.1.2.1)	Yes
33.	Rv0728c	D-3-phosphoglycerate dehydrogenase (EC: 1.1.1.95)	Yes
34.	Rv0505c	Phosphoserine phosphatase (EC: 3.1.3.3)	Yes
35.	Rv0884c	Phosphoserine aminotransferase (EC: 2.6.1.52)	Yes
36.	Rv0409	Acetate kinase (EC: 2.7.2.1)	Yes
37.	Rv0408	Phosphate acetyltransferase (EC: 2.3.1.8)	Yes
38.	Rv3667	Acetyl-CoA synthetase (EC: 6.2.1.1)	Yes
39.	Rv2611c	Lipid A biosynthesis lauroyl acyltransferase (EC: 2.3.1. —)	Yes
40.	Rv0114	D-alpha,beta-D-heptose-1,7-biphosphate phosphatase (EC: 2. —.—.—)	Yes
41.	Rv0113	Phosphoheptose isomerase (EC: 5. —.—.—)	Yes
42.	Rv1315	UDP-N-acetylglucosamine 1-carboxyvinyltransferase (EC: 2.5.1.7)	Yes
43.	Rv0482	UDP-N-acetylenolpyruvoylglucosamine reductase (EC: 1.1.1.158)	Yes
44.	Rv2152c	UDP-N-acetylmuramate-L-alanine ligase (EC: 6.3.2.8)	Yes
45.	Rv2155c	UDP-N-acetylmuramoyl-L-alanyl-D-glutamate synthetase (EC: 6.3.2.9)	Yes
46.	Rv2157c	UDP-N-acetylmuramoylalanyl-D-glutamyl-2,6-diaminopimelate-D-alanyl-D-alanyl ligase MurF	Yes
47.	Rv2156c	Phospho-N-acetylmuramoyl-pentapeptide-transferase (EC: 2.7.8.13)	Yes
48.	Rv2153c	Undecaprenyldiphospho-muramoylpentapeptide beta-N-acetylglucosaminyltransferase (EC: 2.4.1.227)	Yes
49.	Rv2911	D-alanyl-D-alanine carboxypeptidase (EC: 3.4.16.4)	No
50.	Rv2981c	D-alanyl-alanine synthetase A (EC: 6.3.2.4)	Yes
51.	Rv2136c	Undecaprenyl pyrophosphate phosphatase (EC: 3.6.1.27)	Yes
52.	Rv2911	D-alanyl-D-alanine carboxypeptidase (EC: 3.4.16.4)	No
53.	Rv2158c	UDP-N-acetylmuramoylalanyl-D-glutamate-2,6-diaminopimelate ligase (EC: 6.3.2.13)	Yes
54.	Rv2157c	UDP-N-acetylmuramoylalanyl-D-glutamyl-2,6-diaminopimelate-D-alanyl-D-alanyl ligase MurF	Yes
55.	Rv2156c	Phospho-N-acetylmuramoyl-pentapeptide-transferase (EC: 2.7.8.13)	Yes
56.	Rv2153c	Undecaprenyldiphospho-muramoylpentapeptide beta-N-acetylglucosaminyltransferase (EC: 2.4.1.227)	Yes
57.	Rv3910	Transmembrane protein	Yes
58.	Rv0016c	Penicillin-binding protein PbpA	Yes
59.	Rv2163c	Penicillin-binding membrane protein PbpB	Yes
60.	Rv2911	D-alanyl-D-alanine carboxypeptidase (EC: 3.4.16.4)	No

**Table 3:** Shows function of all Essential proteins.

S. no.	Accession. no.	Celllular component	Biological process	Molecular function
1	Rv1820	Not known	Branched chain family amino acid biosynthetic process	Acetolactate synthase activity, magnesium ion binding, thiamine pyrophosphate binding
2.	Rv0951	Cell wall, cytosol	Growth, tricarboxylic acid cycle	ATP binding, metal ion binding, succinate-CoA ligase (ADP-forming) activity
3.	Rv2987c	Plasma membrane, 3-isopropylmalate dehydratase complex	Growth, leucine biosynthetic process	3-Isopropylmalate dehydratase activity
4.	Rv1475c	Cell wall, cytosol, extracellular region, plasma membrane	Growth, response to iron ion	4 iron, 4 sulfur cluster binding, aconitate hydratase activity, iron-responsive element binding
5.	Rv0066c	Cytosol, extracellular region, plasma membrane	Tricarboxylic acid cycle	NAD binding, isocitrate dehydrogenase (NADP+) activity, magnesium ion binding, protein homodimerization activity
6.	Rv2454c	Cell wall, cytosol	Oxidation-reduction process	2-Oxoglutarate synthase activity, magnesium ion binding, thiamine pyrophosphate binding
7.	Rv1240	Cytosol, plasma membrane	Glycolysis, malate metabolic process, tricarboxylic acid cycle	L-malate dehydrogenase activity, binding
8.	Rv1098c	Cytosol, extracellular region, plasma membrane	Growth, tricarboxylic acid cycle	Fumarate hydratase activity
9.	Rv0247c	Plasma membrane	Tricarboxylic acid cycle	Electron carrier activity, iron-sulfur cluster binding, succinate dehydrogenase activity
10.	Rv3356c	Extracellular region, plasma membrane	Folic acid-containing compound biosynthetic process, growth, histidine biosynthetic process, methionine biosynthetic process, one-carbon metabolic process, oxidation-reduction process, purine nucleotide biosynthetic process	Binding, methenyltetrahydrofolate cyclohydrolase activity, methylenetetrahydrofolate dehydrogenase (NADP+) activity
11.	Rv0951	Cell wall, cytosol	Growth, tricarboxylic acid cycle	ATP binding. metal ion binding, succinate-CoA ligase (ADP-forming) activity
12.	Rv0904c	Acetyl-CoA carboxylase complex, plasma membrane	Mycolic acid biosynthetic process	ATP binding, acetyl-CoA carboxylase activity, protein binding
13.	Rv0973c	Plasma membrane	Growth	ATP binding, biotin binding, biotin carboxylase activity
14.	Rv1492	Cell wall, cytosol, plasma membrane	Lactate fermentation to propionate and acetate, propionate metabolic process, methylmalonyl pathway	Cobalamin binding, metal ion binding, methylmalonyl-CoA mutase activity
15.	Rv3667	Cell wall, plasma membrane	Not known	AMP binding, ATP binding, acetate-CoA ligase activity
16.	Rv0409	Cytoplasm	Organic acid metabolic process	ATP binding, acetate kinase activity
17.	Rv0408	Cytoplasm, extracellular region	Not known	Phosphate acetyltransferase activity
18.	Rv0243	Cytosol, plasma membrane	Growth of symbiont in host cell	Acetyl-CoA C-acyltransferase activity
19.	Rv0860	Cytosol, plasma membrane	Fatty acid metabolic process, oxidation-reduction process	Coenzyme binding, oxidoreductase activity
20.	Rv3667	Cell wall, plasma membrane	Not known	AMP binding, ATP binding, acetate-CoA ligase activity
21.	Rv1023	Cell surface, extracellular region, phosphopyruvate hydratase complex, plasma membrane	Glycolysis, growth	Magnesium ion binding, phosphopyruvate hydratase activity
22.	Rv1240	Cytosol, plasma membrane	Glycolysis, malate metabolic process, tricarboxylic acid cycle	L-malate dehydrogenase activity, binding
23.	Rv0070c	Not known	Not known	Not known
24.	Rv2205c	Not known	Organic acid phosphorylation	Glycerate kinase activity
25.	Rv0761c	Oxidation-reduction process	Cytoplasm, plasma membrane	alcohol dehydrogenase (NAD) activity, zinc ion binding
26.	Rv0489	Plasma membrane	Glycolysis	Phosphoglycerate mutase activity
27.	Rv0363c	Extracellular region, plasma membrane	Glycolysis, protein homotetramerization	Fructose-bisphosphate aldolase activity, zinc ion binding
28.	Rv2029c	Not known	Carbohydrate metabolic process	Kinase activity, phosphotransferase activity, alcohol group as acceptor
29.	Rv1908c	Not known	Hydrogen peroxide catabolic process, oxidation-reduction process, response to antibiotic	Catalase activity, heme binding
30.	Rv0070c	Not Known	Not Known	Not known
31.	Rv0728c	Not Known	Oxidation-reduction process	NAD binding, phosphoglycerate dehydrogenase activity
32.	Rv0505c	Integral to plasma membrane	Not Known	Metal ion binding, phosphatase activity
33.	Rv0884c	Cytoplasm, extracellular region, plasma membrane	L-serine biosynthetic process, growth, pyridoxine biosynthetic process	O-phospho-L-serine: 2-oxoglutarate aminotransferase activity, pyridoxal phosphate binding
34.	Rv0409	Cytoplasm	Organic acid metabolic process	ATP binding, acetate kinase activity
35.	Rv0408	Cytoplasm, extracellular region	Not known	Phosphate acetyltransferase activity
36.	Rv3667	Cell wall, plasma membrane	Not known	AMP binding, ATP binding, acetate-CoA ligase activity
37.	Rv2611c	Integral to membrane, plasma membrane	Glycolipid biosynthetic process, growth, lipopolysaccharide core region biosynthetic process	Acyltransferase activity
38.	Rv0114	Cytoplasm	Carbohydrate metabolic process, histidine biosynthetic process	Histidinol-phosphatase activity
39.	Rv0113	Cytoplasm	Carbohydrate metabolic process	D-sedoheptulose 7-phosphate isomerase activity, metal ion binding, sugar binding
40.	Rv1315	Cytoplasm	UDP-N-acetylgalactosamine biosynthetic process, cell cycle, cell division, cellular cell wall organization, growth, peptidoglycan biosynthetic process, regulation of cell shape	UDP-N-acetylglucosamine 1-carboxyvinyltransferase activity
41.	Rv0482	Cytoplasm	Cell cycle, cell division, cellular cell wall organization, oxidation-reduction process, peptidoglycan biosynthetic process, regulation of cell shape	UDP-N-acetylmuramate dehydrogenase activity, flavin adenine dinucleotide binding
42.	Rv2152c	Cytoplasm	Cell cycle, cell division, cellular cell wall organization, growth, peptidoglycan biosynthetic process, regulation of cell shape	ATP binding, UDP-N-acetylmuramate-L-alanine ligase activity
43.	Rv2155c	Cytosol	Cell cycle, cell division, cellular cell wall organization, growth, peptidoglycan biosynthetic process, regulation of cell shape	ATP binding, UDP-N-acetylmuramoylalanine-D-glutamate ligase activity, protein binding
44.	Rv2157c	Cytoplasm	Cell cycle, cell division, cellular cell wall organization, growth, peptidoglycan biosynthetic process, regulation of cell shape	ATP binding, UDP-N-acetylmuramoyl-tripeptide-D-alanyl-D-alanine ligase activity, UDP-N-acetylmuramoylalanyl-D-glutamyl-2,6-diaminopimelate-D-alanyl-D-alanine ligase activity
45.	Rv2156c	Integral to membrane, plasma membrane	Cell cycle, cell division, cellular cell wall organization, growth, peptidoglycan biosynthetic process, regulation of cell shape	Phospho-N-acetylmuramoyl-pentapeptide-transferase activity
46.	Rv2153c	Plasma membrane	Cell cycle, cell division, cellular cell wall organization, growth, regulation of cell shape, UDP-N-acetylgalactosamine biosynthetic process, lipid glycosylation, peptidoglycan biosynthetic process	Carbohydrate binding, undecaprenyldiphospho-muramoylpentapeptide beta-N-acetylglucosaminyltransferase activity
47.	Rv2981c	Cell wall, cytoplasm, plasma membrane	Cellular cell wall organization, growth, peptidoglycan biosynthetic process, regulation of cell shape	ATP binding, D-alanine-D-alanine ligase activity, metal ion binding
48.	Rv2136c	Integral to membrane, plasma membrane	Cellular cell wall organization, peptidoglycan biosynthetic process, regulation of cell shape, dephosphorylation, response to antibiotic, response to nitrosative stress	Undecaprenyl-diphosphatase activity
49.	Rv2158c	Cytosol, plasma membrane	Cell cycle, cell division, cellular cell wall organization, peptidoglycan biosynthetic process, regulation of cell shape	ATP binding, UDP-N-acetylmuramoylalanyl-D-glutamate-2,6-diaminopimelate ligase activity
50.	Rv2157c	Cytoplasm	Cell cycle, cell division, cellular cell wall organization, growth, peptidoglycan biosynthetic process, regulation of cell shape	ATP binding, UDP-N-acetylmuramoyl-tripeptide-D-alanyl-D-alanine ligase activity, UDP-N-acetylmuramoylalanyl-D-glutamyl-2,6-diaminopimelate-D-alanyl-D-alanine ligase activity
51.	Rv2156c	Integral to membrane, plasma membrane	Cell cycle, cell division, cellular cell wall organization, growth, peptidoglycan biosynthetic process, regulation of cell shape	Phospho-N-acetylmuramoyl-pentapeptide-transferase activity
52.	Rv2153c	Plasma membrane	Cell cycle, cell division, cellular cell wall organization, growth, peptidoglycan biosynthetic process, regulation of cell shape, UDP-N-acetylgalactosamine biosynthetic process	Carbohydrate binding, undecaprenyldiphospho-muramoylpentapeptide beta-N-acetylglucosaminyltransferase activity
53.	Rv3910	Integral to plasma membrane	Not known	Not known
54.	Rv0016c	Cell septum, cytosol, integral to membrane, plasma membrane	Cellular cell wall organization, peptidoglycan biosynthetic process, regulation of cell shape	Penicillin binding, transferase activity
55.	Rv2163c	Extracellular region	Growth, peptidoglycan-based cell wall biogenesis	Penicillin binding, protein binding

## Appendix

See Tables 1, 2, and 3.
